# Rheological, physicochemical, and microstructural properties of asphalt binder modified by fumed silica nanoparticles

**DOI:** 10.1038/s41598-021-90620-w

**Published:** 2021-06-01

**Authors:** Goshtasp Cheraghian, Michael P. Wistuba, Sajad Kiani, Andrew R. Barron, Ali Behnood

**Affiliations:** 1 Braunschweig Pavement Engineering Centre, Technische Universität Braunschweig, Braunschweig, Germany; 2 Energy Safety Research Institute (ESRI), Swansea University, Bay Campus, Swansea, SA1 8EN UK; 3 Department of Chemistry and Department of Materials Science and Nanoengineering, Rice University, Houston, TX 77005 USA; 4 Faculty of Engineering, Universiti Teknologi Brunei, Gadong, Brunei Darussalam; 5 Lyles School of Civil Engineering, Purdue University, 550 Stadium Mall, West Lafayette, IN 47907-2051 USA

**Keywords:** Civil engineering, Nanoscale materials, Nanoparticles, Nanoscience and technology

## Abstract

Warm mix asphalt (WMA) is gaining increased attention in the asphalt paving industry as an eco-friendly and sustainable technology. WMA technologies are favorable in producing asphalt mixtures at temperatures 20–60 °C lower in comparison to conventional hot mix asphalt. This saves non-renewable fossil fuels, reduces energy consumption, and minimizes vapors and greenhouse gas emissions in the production, placement and conservation processes of asphalt mixtures. At the same time, this temperature reduction must not reduce the performance of asphalt pavements in-field. Low aging resistance, high moisture susceptibility, and low durability are generally seen as substantial drawbacks of WMA, which can lead to inferior pavement performance, and increased maintenance costs. This is partly due to the fact that low production temperature may increase the amount of water molecules trapped in the asphalt mixture. As a potential remedy, here we use fumed silica nanoparticles (FSN) have shown excellent potential in enhancing moisture and aging susceptibility of asphalt binders. In this study, asphalt binder modification by means of FSN was investigated, considering the effects of short-term and long-term aging on the rheological, thermal, and microstructural binder properties. This research paves the way for optimizing WMA by nanoparticles to present enhanced green asphalt technology.

## Introduction

According to the Paris Agreement and in order to decrease the impacts and risks of climate change, there is need to reduce greenhouse gas emissions significantly and to keep global average temperature increase below 2 °C^1,2^. Road infrastructures account for approximately 22%, 28%, and 63% of global carbon dioxide emissions, world energy consumption, and total transportation sector-related greenhouse gas emissions, respectively^3^. In comparison to conventional hot mix asphalt technology, the warm mix asphalt (WMA) technology enables asphalt mix production at temperatures of approximately 50 °C lower, and hence, it is an up-to-date solution for reducing energy consumption by 35%, greenhouse gas emissions by 20 to 35%, and harmful vapors such as polycyclic aromatic hydrocarbons significantly^4,5^.

WMA is gaining attention in the asphalt paving industry as an eco-friendly and sustainable technology. WMA technologies reduce production temperatures by 20 to 60 °C in comparison to conventional hot mix asphalt. WMA reduces energy consumption while also minimizing vapors and greenhouse gas emissions for the production of asphalt mixtures^5^. In addition, WMA is produced at a lower temperature condition than conventional asphalt mixtures, thus thermal and oxidative aging in WMA is lower than the conventional type^6^. Nonetheless, aging of the WMA mixture may be a disadvantage limiting in-field performance. Thus, the investigation of anti-aging techniques and modifications is a challenging topic in the field of modern asphalt pavement engineering^6,7^. The warm mix asphalt technologies can be divided into three categories by use of synthetic or organic additives which ultimately affect the level of temperature reduction: chemical, organic additives, and foaming techniques^5^. Some research studies postulate, that wax-based warm mix additives age less then chemical-based warm mix additives, and that WMA with wax-based warm mix additives have better rheological and mechanical properties in higher oxidation levels^8,9^.

Generally, asphalt aging includes thermo oxidative short-term aging, and thermal and ultraviolet long-term aging. Volatilization, oxidation, and steric hardening all play key roles. While oxidation and volatilization are the result changes in molecules, steric hardening is due to molecular rearrangement^10^. Temperature, ultraviolet radiation, and oxygen are the three major factors that make the asphalt binder stiffer and more brittle, owing to the decreasing content of soft aromatic components, and the increased content of resins and quasi-solid asphaltenes^11^.

Due to the significant reduction in temperature when producing WMA, any undesired changes in binder properties in consequence of aging are reduced, however, it still exists as a problem. Furthermore, the temperature reduction in temperature may reduce asphalt mixture performance. Moisture susceptibility is also considered as a remarkable drawback of WMA pavements, causing inferior pavement performance, and increasing maintenance costs. This is partly due to the fact that low production temperature may increase the level of water molecules trapped in the asphalt mixture^12,13^.

As a solution to tackle the aging and moisture issue in WMA mixtures, the asphalt binder can be modified by anti-aging additives. Among a vast variety of possible anti-aging additives, the use of nanoparticles (NPs) is considered one of the most efficient solutions. The latest research studies promote NPs-modified binders as beneficial in terms of life cycle costs, technical performance, and environmental impacts^14,15^. NPs can improve chemical and mechanical binder performance significantly. Chemical composition, shape, and surface area to volume all contribute to the unique physical properties of NPs when compared to conventional materials^16,17^. Various types of NPs, such as silica NPs, zinc NPs, TiO_2_ NPs, clay NPs, nano fibers, and nano polymer composites have been reported in successful binder modification^18,19^. Inter alia, rheological performance of binder and asphalt mixture were all improved with silica NPs, fatigue strength of binder was increased with titanium dioxide NPs, and mechanical and thermal stabilities of asphalt mixtures were improved by clay NPs^20–23^. Silica NPs possess important advantages such as being non-toxic, non-photocatalytic, and inorganic^24–26^. Silica families (based on SiO_2_ arrangements) were reported to be one of the best inorganic NPs in improving binder aging properties^27,28^. Yao et al.^29^ presented a detailed investigation of nano-silica-modified asphalt binders. Based on their results, viscosity and complex modulus were slightly decreased, while fatigue and rutting resistance were improved after short-term aging. Saltan et al.^30^ reported that nano-silica-modified binder has higher resistance to thermal aging, ultimately leading to increased durability of asphalt pavements. In addition, it was also observed that rutting and fatigue performance could be enhanced when using 0.3 wt.-% of silica NPs. Ghanoon and Tanzadeh^31^ confirmed that rutting resistance and creep recovery were improved.

Guo et al.^32^ reported on fumed silica nanoparticles (FSN) on a large scale. FSN represent a synthetically produced nanomaterial that is composed of an amorphous structure in a nano size scale and a large surface area. Fumed silica are low-cost nanomaterials, non-toxic, and can be used as inorganic shielding^33,34^. It was reported, that FSN has potential to improve bitumen mechanical and rheological properties^20,35,36^. However, FSN as a nanoparticle is yet to be used in WMA.

**Figure 1:**
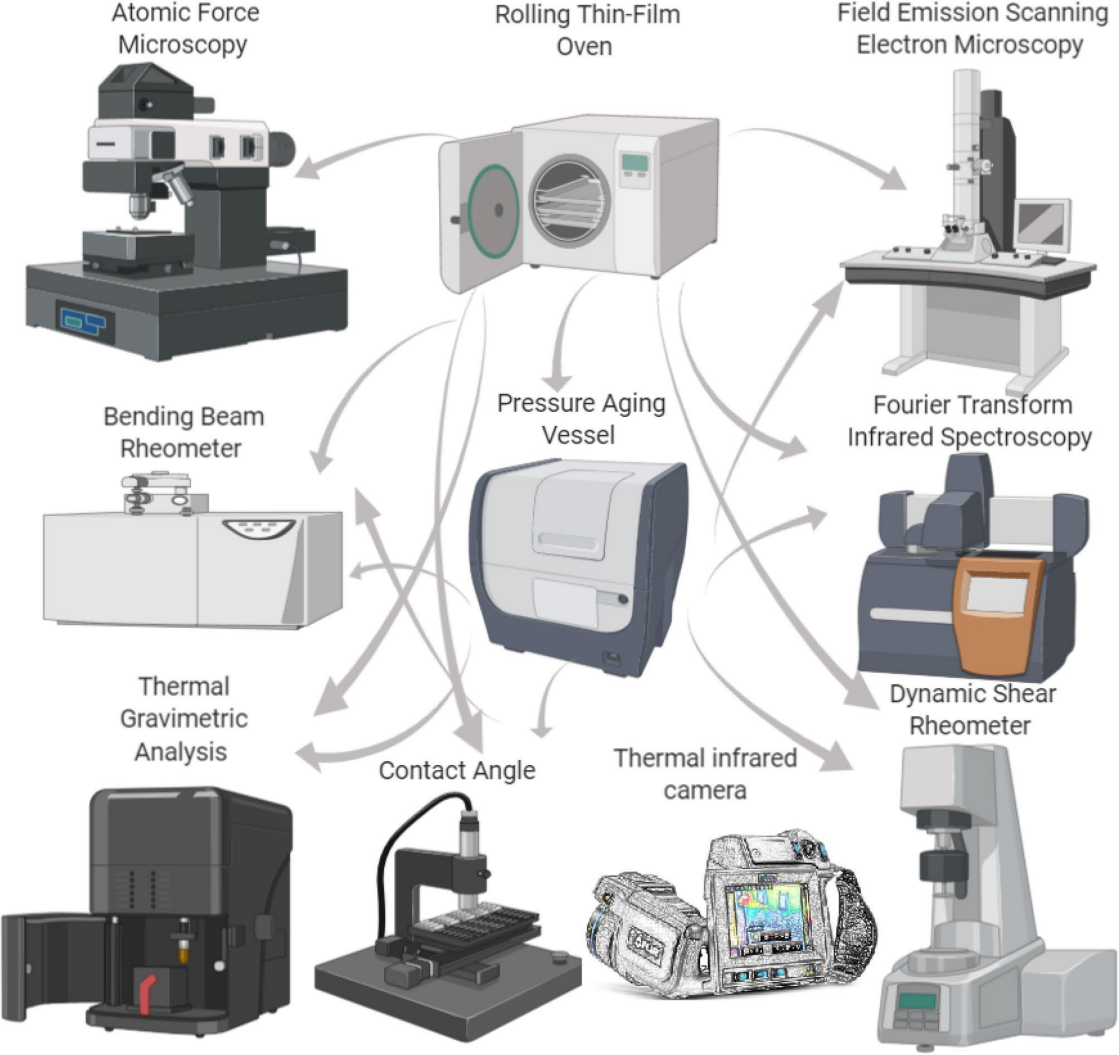
Schematic overview on experimental techniques applied in this study.

In the present study, the potential of FSN was screened as an anti-aging agent specially to be use for WMA binder modification. The characteristics and properties of FSN-modified binder samples were investigated in different concentrates and conditions by means of Dynamic Shear Rheometer (DSR), Field Emission Scanning Electron Microscopy (FESEM), Thermal Gravimetric Analysis (TGA), Contact Angle (CA), Bending Beam Rheometer (BBR), Atomic Force Microscopy (AFM) and Fourier Transform Infrared Spectroscopy (FTIR) tests. The Rolling Thin-Film Oven (RTFO) and Pressure Aging Vessel (PAV) tests were used to simulate thermal-oxidative binder aging for short-term and long-term conditions separately. Figure 1 schematically illustrates the experimental techniques applied in this study.

## Results and discussion

### Dynamic viscoelastic properties

#### Complex modulus and phase angle

**Figure 2:**
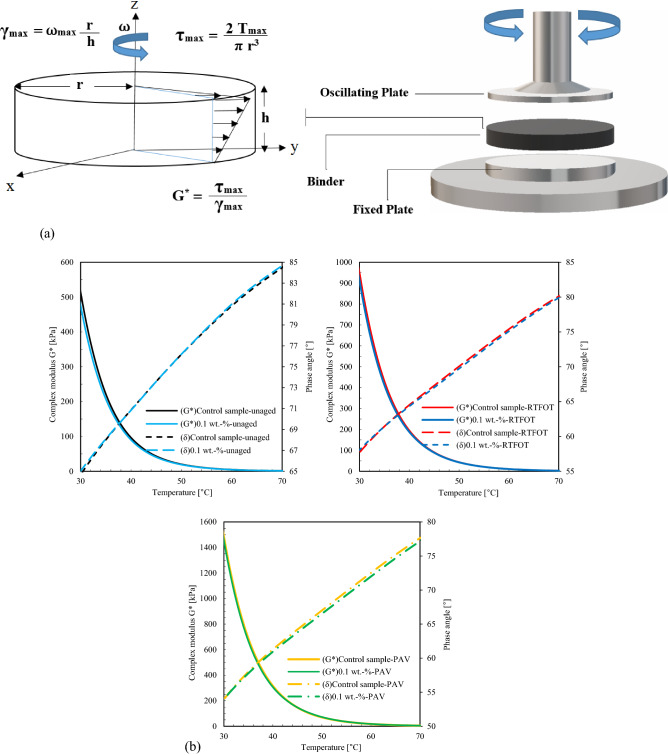

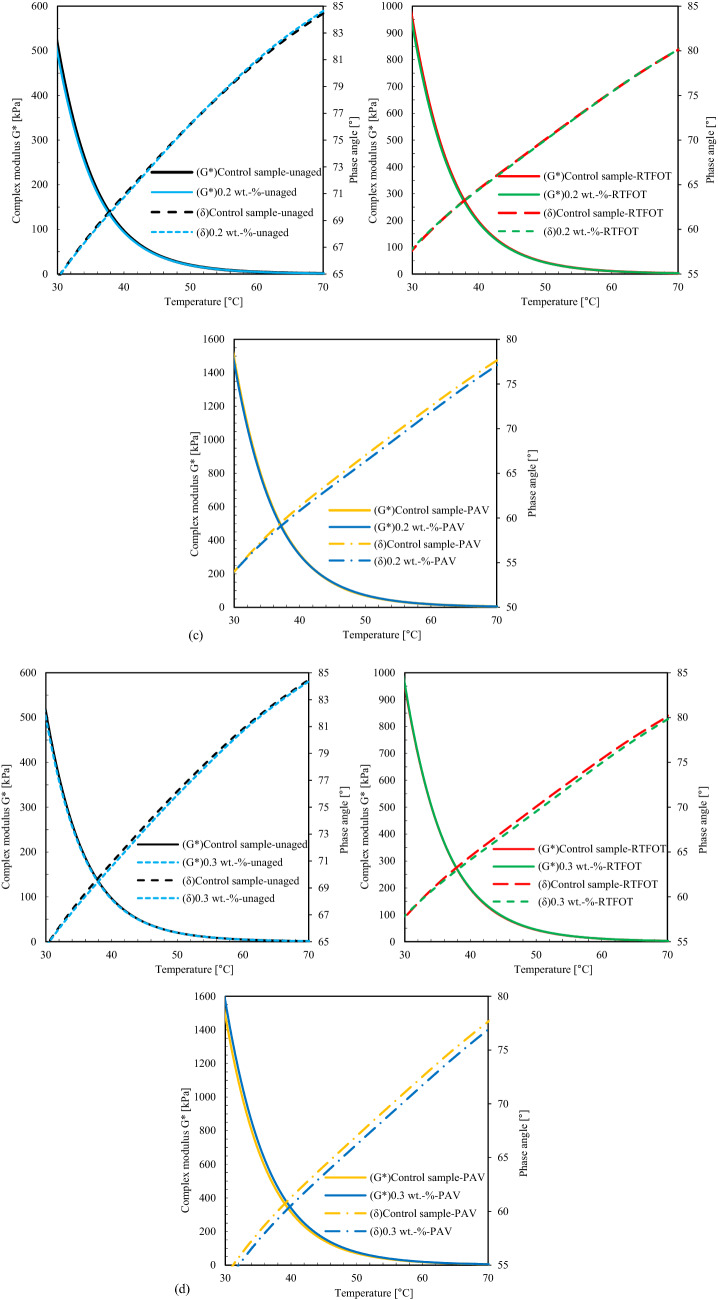
Analysis of Dynamic Shear Rheometer tests: (**a**) Schematic of rotational plate viscosity test. Complex modulus and phase angle of binders modified by fumed silica nanoparticles (FSN) before and after laboratory aging with (**b**) 0.1 wt.-%, (**c**) 2 wt.-%, and (**d**) 3 wt.-% of FSN.

Rheological binder characteristics in terms of complex modulus and phase angle were studied based on Dynamic Shear Rheometer (DSR) tests (AASHTO T 315). The dynamic rheological properties have measured in the viscoelastic region. Figure 2a illustrates schematically the equations to calculate the rotational plate viscosity from DSR tests. DSR tests were run with an oscillating plate of 25 mm in diameter and a 1 mm gap distance between the rotated and the fixed plate, where h is the specimen height (m), r is the specimen radius (m), T_max_ is the maximum applied torque (N-m), and ⍵_max_ is the maximum rotation angle (radians). The complex modulus (G*) was measured based on shear strain (γ) and shear stress (τ)^37^.

Figure 2b-d shows the effects of aging and different concentrates of fumed silica nanoparticles (FSN) on the complex modulus. Samples modified with FSN have higher complex modulus, and sample S10 has highest deformation resistance before aging. The aging period significantly increases in complex modulus, while for FSN-modified samples this effect is decreased. A concentration of 0.1 wt.-% of FSN shows the most distinct impact on both short-term and long-term aging resistance. The optimum range of FSN concentration in the binder was found between 1 and 2 wt.-%.

Figure 2 shows the different phase angles observed. Phase angles increase with the addition of FSN, which is explained by a transformation of the binder properties from elastic to viscous^38^. For samples S7 and S11, the smallest transformation levels are obtained, for short-term and long-term aging, respectively, resulting in smallest phase angle after aging. The viscoelastic transformation changes the rheological properties of samples; the ranking of them were found to be 0.3 wt.-% FSN < 0.1 wt.-% FSN < 0.2 wt.-% FSN for short and long-term aging. These outcomes indicate that FSN-modification improves binder aging resistance, and the amount of 0.1 wt.-% can be considered as optimum.

#### Resistance to permanent deformation

**Figure 3:**
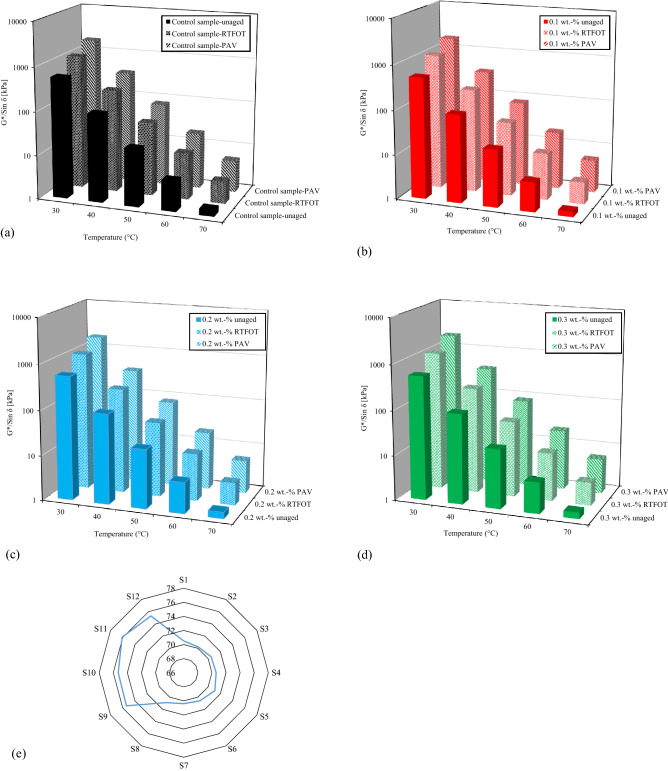
Analysis of Dynamic Shear Rheometer tests—rutting resistance of binder samples before and after aging: (**a**) control sample, (**b**) 0.1 wt.-% fumed silica nanoparticles (FSN), (**c**) 0.2 wt.-% FSN, and (**d**) 0.3 wt.-% FSN. (**e**) Binder threshold temperatures (°C) for rutting resistance considering different concentrations of FSN before and after aging, G*/sin δ = 1 kPa for before aging and G*/sin δ = 2.2 kPa for after aging.

Rutting resistance was analyzed based on DSR standard SHRP-A-369. Rutting parameters in terms of DSR-limiting parameter G*/sin δ between 30 and 70 °C before and after aging are presented in Fig. 3. The results demonstrate that FSN improved the resistance to permanent deformation. Samples 6 and 10 with 0.1 wt.-% FSN after aging showed the most distinct improvement. However, rutting parameters after long-term aging were found to be approximately the same across the entire temperature range. It appears that a specific concentration of FSN satisfies any rutting requirements. This result is in agreement with findings from other researches on nano-reinforced binders^20,39–42^.

In this work, rutting resistance was evaluated by considering a threshold temperature for rutting^39^. According to SHRP-A-369 standard, a value of G*/sin δ  = 1 kPa is considered for unaged samples, and a value of 2.2 kPa for aged samples, as represented in Fig. 3e. It is obvious, that FSN decreased binder rutting resistance before aging, while after aging, the threshold temperature decreased with the addition of FSN. Moreover, a concentration of 0.1 wt.-% of FSN showed better performance after short-term aging process than higher concentrations. Furthermore, after long term aging, considerable differences were not found.

### Low-temperature performance

**Figure 4:**
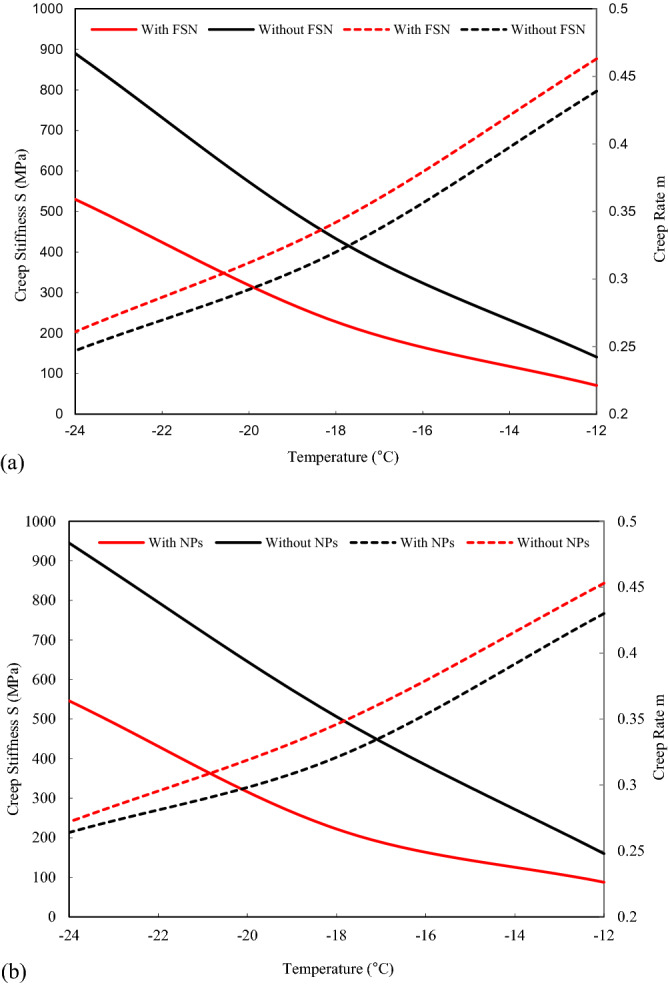
Analysis of Bending Beam Rheometer tests: Creep Stiffness (S) and Creep Rate (m) of binder modified with fumed silica nanoparticles (FSN) (**a**) before and (**b**) after long-term aging at different temperatures.

Low temperature performance properties were studied using Bending Beam Rheometer tests (AASHTO T 313). Creep Stiffness (S) and Creep Rate (m) for FSN-modified binders were always calculated at three different temperatures (Fig. 4), i. e. − 12 °C, − 18 °C and − 24 °C. It was observed that S increased with the aging degree with reduction in temperature. In addition, FSN decreased the level of S and increased the level of m, which was interpreted as an increase in cracking resistance, as modified binder samples have suitable elasticity properties at low temperatures^43,44^. Sample S10 with 0.1 wt.-% of FSN showed best performance.

### Thermal and spectroscopy analysis

**Figure 5:**
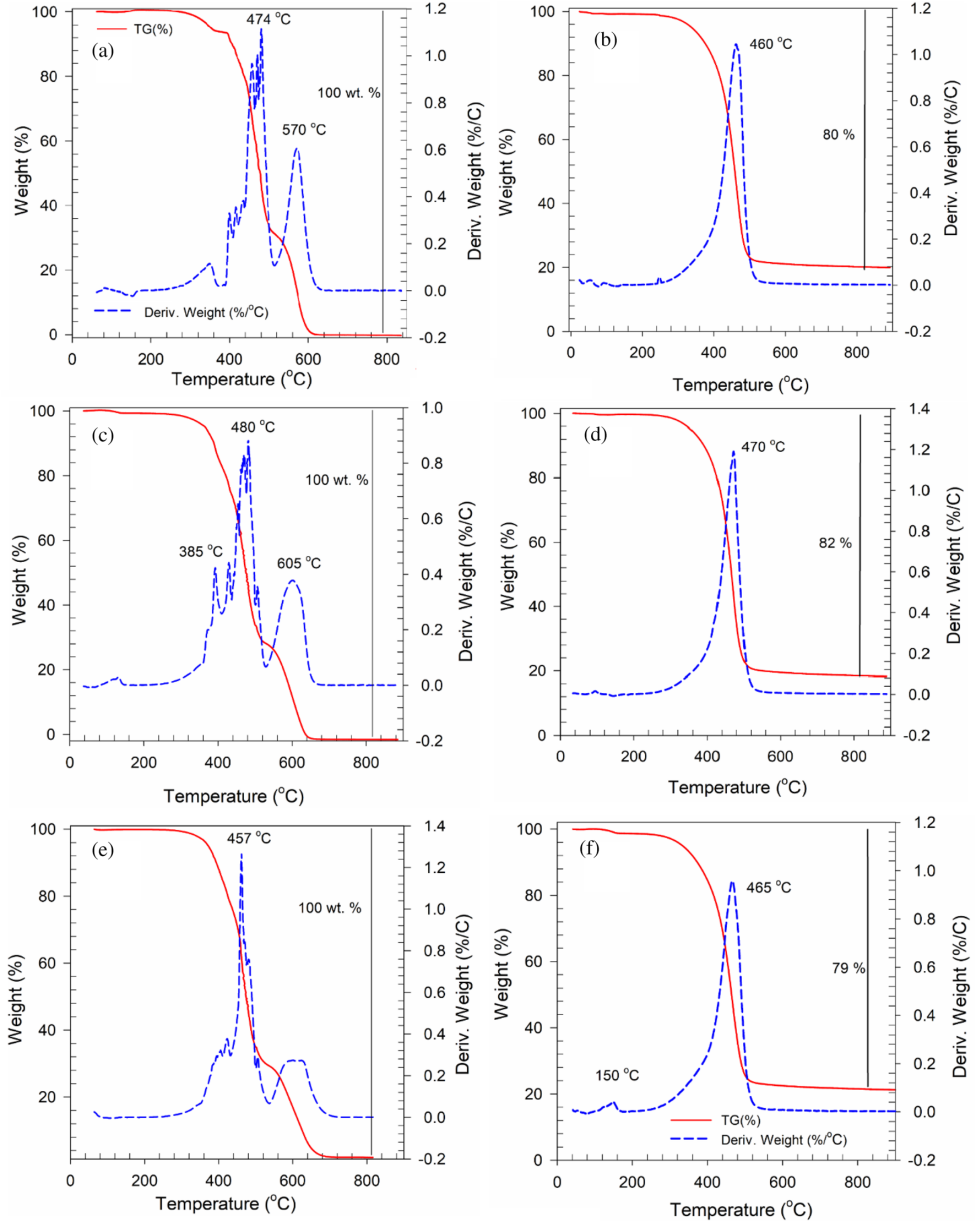
Thermal Gravimetric Analysis (TGA) and Derivative Thermo-Gravimetry (DTG) curve: (**a**) unaged binder, (**b**) unaged binder modified by FSN, (**c**) short-term aged binder, (**d**) FSN-modified binder after short-term aging, (**e**) long-term aged binder, and (**f**) FSN-modified binder after long-term aging.

Fourier transform infrared spectroscopy (FTIR) was carried out to assess the surface functionalities of binder samples in the range of 650–4000 cm^−1^ using a Thermo Scientific Nicolet iS10 FT-IR Spectrometer. Thermogravimetric analysis (TGA) was applied to measure the loss of sample weight while the temperature was increased to 900 °C. In order to study the thermal decomposition pattern and thermal stability, Derivative Thermo-Gravimetry (DTG) analysis and Thermal Gravimetric Analysis (TGA) were conducted (Fig. 5).

Suitable thermal stability was found for FSN-modified binder samples. There were three stages for thermal decomposition identified: mass loss is in a temperature range of 200 to 360 °C, then again in a range of 370 to 490 °C, and finally around 540 °C. For control samples in the range 480–580 °C, a region was found related to the decomposition of heteroatom bonds, functional groups, and dehydrogenation and oxidation of resins. After this step, char and asphaltenes are created in polymerization/cracking process. These reactions were found to provoke weight loss of binders^45–47^. In FSN-modified binder samples, the final decomposition occurred earlier than for control samples, which was attributed to the presence of FSN. A surplus of 18–21% residue of carbon was recorded for FSN-modified binder samples at 800 °C, when mass loss was highest, while for the control samples 0% carbon residue was recorded at the same temperature. The lower weight loss can be interpreted as an indicator for lower evaporation and higher heat stability of the FSN-modified binders^48^. A significant mass loss was observed for aged binders beyond 200 °C. Note, that this temperature is much higher than the required operational temperature for hot mix asphalt. Hence, it was concluded that FSN in binders have acceptable thermal performance.

**Figure 6:**
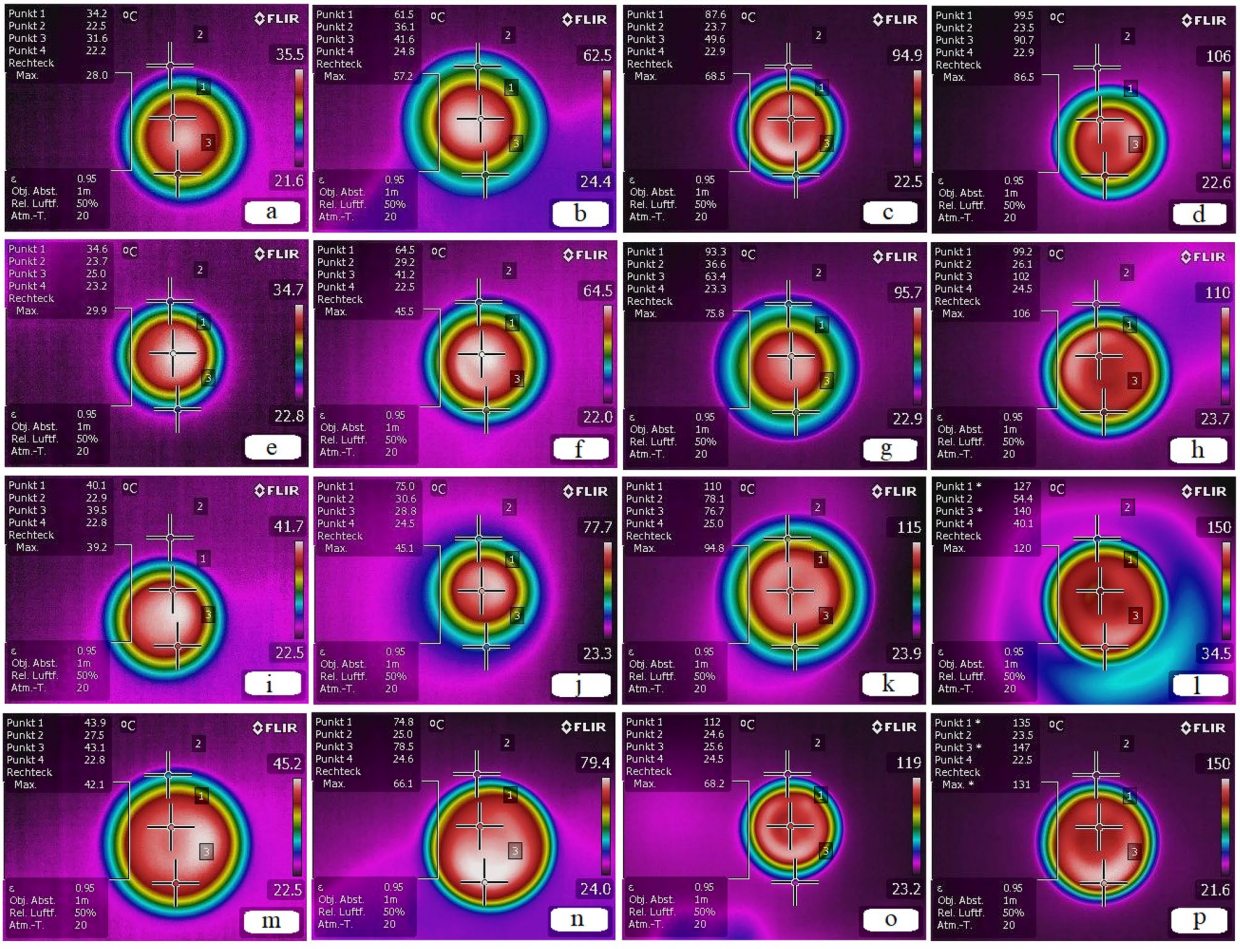
Infrared thermal image evaluation of asphalt binder specimens: (**a**-**d**) warm mix asphalt (WMA) additive and FSN, (**e**–**h**) WMA additive and without FSN, (**i**-**l**) FSN and without WMA additive, and (**m**-**p**) without WMA additive and without FSN; in different thermal zones: (**a**,**e**,**I**,**m**) 30 s, (**b**,**f**,**j**,**n**) 60 s, (**c**,**g**,**k**,**o**) 90 s, and (**d**,**h**,**l**,**p**) 120 s. The diameter of the specimen is 18 mm.

Figure 6 shows the temperature distribution (infrared image) on the surface of the samples at different times. The temperature distribution is represented by the color variation of binder samples, where darker and higher colors represent lower and higher temperatures, respectively. The surface temperature evolution during 120 s is represented in Fig. 6. The temperature distribution of samples was calculated for three surface points^49,50^. At low temperature (Fig. 6a,e,i,m) heat distribution was found to be uniform, but after 120 s the temperature increased from 34.6 to 102 °C and from 43.9 to 147 °C for samples with and without WMA additive, respectively. Maybe, here limited heat loss should be considered as an experimental error.

A linear growth trend in surface temperature and heating rates of samples without WMA additive was identified. As seen in Fig. 6h,p, samples with WMA additive released heat uniformly in lower temperature range, while samples without WMA additive created heat accumulation. This leads to the conclusion, that samples with WMA additives at a lower heat level present a more suitable temperature distribution then samples without WMA additive. Figure 6c-h,m-p shows that the release heat rate of asphalt binders with and without FSN are 0.66 and 0.68 °C/s, respectively. This indicates that FSN has slightly better performance in the temperature distribution. In addition, this observation may be linked with recent findings from Badroodi et al.^51^, indicating that FSN support self-healing in asphalt binders.

**Figure 7:**
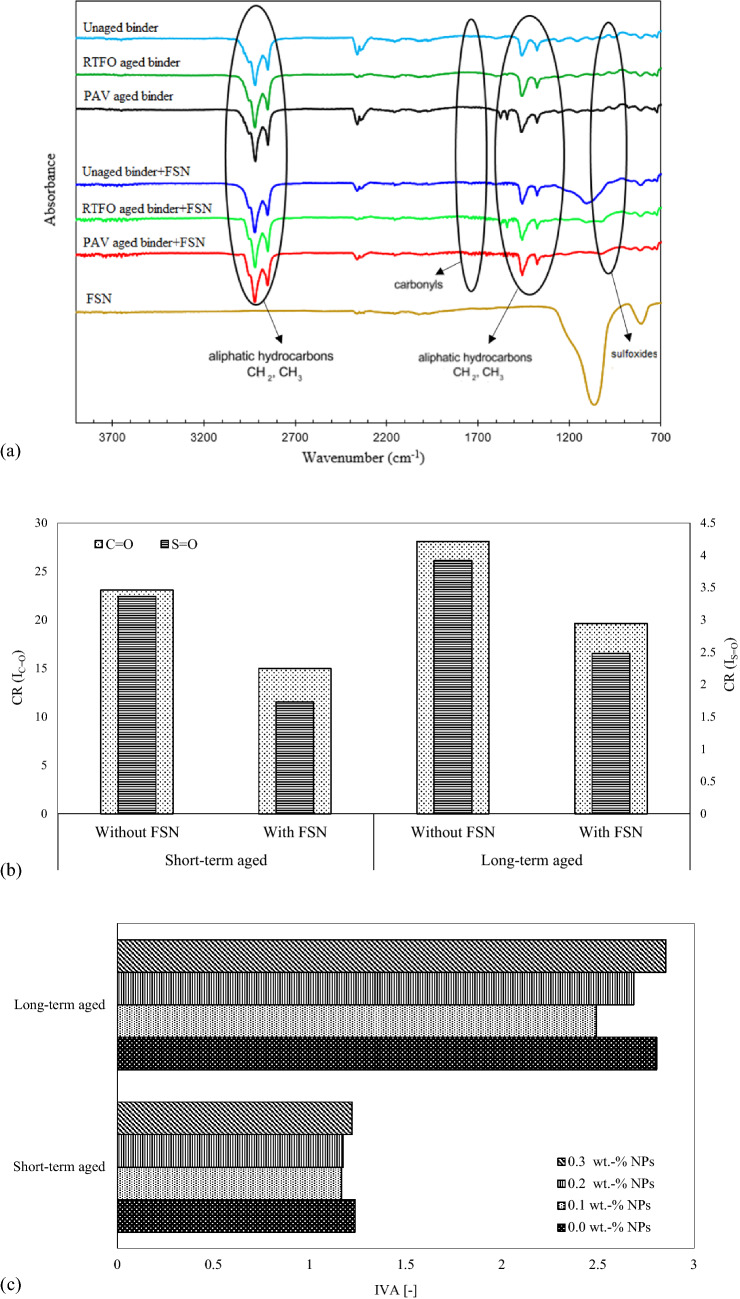
(**a**) Fourier Transform Infrared Spectroscopy (FTIR) spectra of the binder samples in the wavenumber range from 700 to 4000 cm^1^ for different aging conditions. (**b**) Aging indexes based on carbonyl and sulfoxide groups at different aging conditions. (**c**) Index of viscosity aging (IVA) of control and modified binders.

Figure 7a shows the FTIR spectra of FSN-modified and control binder samples. The infrared spectrum of chemical bonds were found to be reflected in various locations. The sulfoxides, the carbonyls, the aromatics, the polyaromatics, and the aliphatic hydrocarbons groups are created by oxidative aging from carbon–carbon or carbon–hydrogen bonds. In order to calculate the change of chemical bonding, the following equations were used according to^52,53^:

1 $${\text{I}}_{{{\text{CH}} = {\text{CH}}}} = \frac{{{\text{Area}}\;{\text{of}}\;{\text{carbonyl}}\;{\text{band}}\;{\text{centered}}\;{\text{around }}\;966\;{\text{cm}}^{ - 1} }}{{\sum {{\text{Area}}\;{\text{of}}\;{\text{spectral}}\;{\text{bands}}\;{\text{around }}\;2000 \;{\text{and}}\; 600 \;{\text{cm}}^{ - 1} } }}$$

2 $${\text{I}}_{{\text{S = O}}} = \frac{{{\text{Area}}\;{\text{of }}\;{\text{carbonyl}}\;{\text{band }}\;{\text{centered}}\;{\text{around}}\;{1030}\;{\text{cm}}^{{ - {1}}} }}{{\sum {{\text{Area}}\;{\text{of }}\;{\text{spectral}}\;{\text{ bands}}\;{\text{ around}}\;{ 2000}\;{\text{ and}}\;{ 600 }\;{\text{cm}}^{{ - {1}}} } }}$$

3 $${\text{I}}_{{{\text{C - H}}\;{\text{of}}\;{\text{CH}}_{{3}} }} = \frac{{{\text{Area}}\;{\text{of}}\;{\text{carbonyl}}\;{\text{band}}\;{\text{centered}}\;{\text{around}}\;{1376 }\;{\text{cm}}^{ - 1} }}{{\sum {\text{Area}}\;{\text{of}}\;{\text{spectral}}\;{\text{bands}}\;{\text{around}}\;{2000}\;{\text{and}}\;{600 }\;{\text{cm}}^{ - 1} }}$$

4 $${\text{I}}_{{{\text{C - H of }}\left( {{\text{CH}}_{{3}} } \right){\text{n}}}} = \frac{{{\text{Area}}\;{\text{of}}\;{\text{carbonyl}}\;{\text{band}}\;{\text{centered}}\;{\text{around}}\;{1460 }\;{\text{cm}}^{ - 1} }}{{\sum {\text{Area}}\;{\text{of}}\;{\text{spectral}}\;{\text{bands}}\;{\text{around}}\;{2000}\;{\text{and}}\;{600}\;{\text{cm}}^{ - 1} }}$$

5 $${\text{I}}_{{\text{C = C}}} = \frac{{{\text{Area }}\;{\text{of }}\;{\text{carbonyl}}\;{\text{band}}\;{\text{centered}}\;{\text{around}}\;{1600}\;{\text{cm}}^{ - 1} }}{{\sum {\text{Area}}\;{\text{of}}\;{\text{spectral}}\;{\text{bands}}\;{\text{around}}\;{2000}\;{\text{and}}\;{600}\;{\text{cm}}^{ - 1} }}$$

6 $${\text{I}}_{{\text{C = O}}} = \frac{{{\text{Area of carbonyl band centered around}}\;1690\;{\text{cm}}^{ - 1} }}{{\sum {\text{Area of spectral bands around}}\, 2000\;{\text{and}}\;600\;{\text{cm}}^{ - 1} }}$$

7 $${\text{CR}} = \left( {\frac{{{\text{Index}}\;{\text{of}}\;{\text{bitumen}}\;{\text{after}} - {\text{Index}}\;{\text{of}}\;{\text{bitumen}}\;{\text{before}}}}{{{\text{Index}}\;{\text{of}}\;{\text{bitumen}}\;{\text{before}}}}} \right) \cdot {1}00$$

**Table 1:** Rate changes of chemical bands in different aging conditions.

	Sample	C=O	S=O	C=C	CH=CH	C–H of (CH_2_)n	C–H of CH_3_
Unaged	Without FSN	0.005156	0.06724	0.03503	0.06013	0.1622	0.05853
	With FSN	0.005354	0.06755	0.03384	0.06050	0.1440	0.05040
Short-term aged	Without FSN	0.006701	0.06958	0.03618	0.06051	0.1487	0.05662
	With FSN	0.006299	0.06874	0.03523	0.06074	0.1249	0.05611
Long-term aged	Without FSN	0.007168	0.06998	0.0362	0.06092	0.1324	0.05365
	With FSN	0.006660	0.06927	0.03624	0.06105	0.1306	0.05203

**Figure 8:**
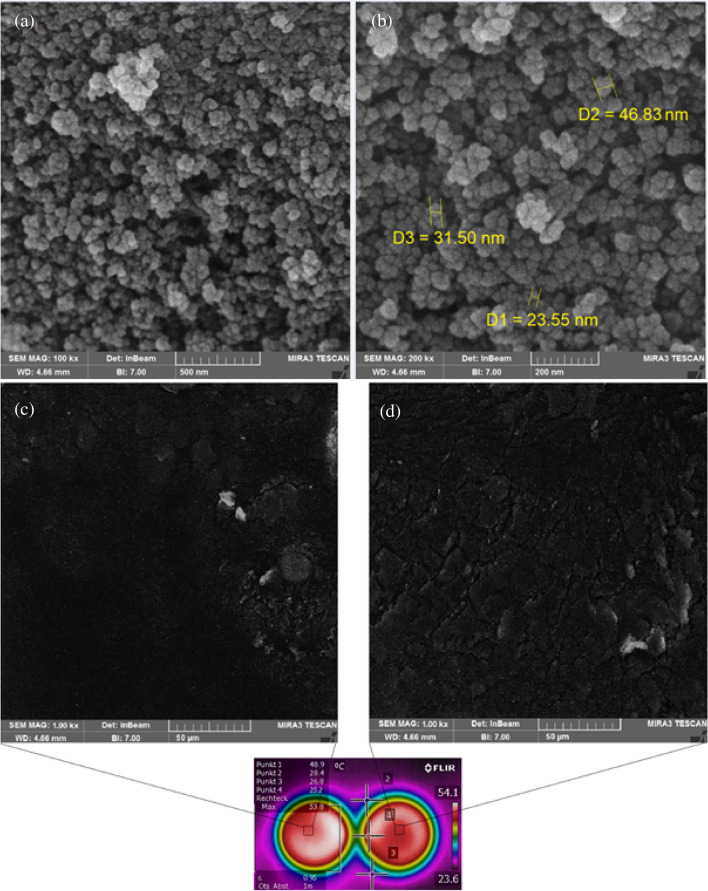
FESEM images of binder modified by fumed silica nanoparticles (FSN) at different magnifications. Average particle sizes of FSN are 33 nm. Uniformly and hexagonal structure shapes created with an average diameter of 100–300 nm. FESEM images of binder modified by FSN (**a**-**c**) unaged, and (**b**-**d**) after aging.

Rate changes of chemical bonds are represented in Table 1 and Fig. 7b. The sulfoxide (S=O) and the carbonyl (C=O) indexes increased during short-term and long-term aging. The carbonyl and sulfoxide indexes decreased for FSN-modified binder samples after aging. Hence, FSN-modification caused a delayed and weakened binder aging. As a consequence of aging asphaltenes are hydrogenated into hydroaromatic hydrocarbons or polycyclic aromatics, and aromatic compounds increase. On the other hand, due to the contrast between aromatic compounds and aliphatic compounds, aliphatic molecules decrease due to aging. Moreover, the ethylene index (CH=CH) increased in FSN-modified binder samples after aging which shows that FSN are a suitable protective against thermal reactions and oxidation^52^. In addition, the WMA additive altered the spectrum range of O–H vibrations (3300–3600 cm^−1^). In other words, FT waxes caused a change in hydrogen bonding between asphalt molecules and reduced the viscosity of the binder^53^.

### Mechanical properties

The index of viscosity aging (IVA) is a measure for aging resistance of asphalt binders, as a low IVA goes together with a high aging resistance^39^. IVA is calculated from the aged binder viscosity and the unaged binder viscosity, according to Eq. (8):

8 $${\text{IVA}} = \frac{{{\text{viscosity}}\;{\text{of}}\;{\text{binder}}\;{\text{after}}\;{\text{aging}} - {\text{viscosity}}\;{\text{of}}\;{\text{unaged}}\;{\text{binder}}}}{{{\text{viscosity}}\;{\text{of}}\;{\text{unaged}}\;{\text{binder}}}}$$

Figure 7c illustrates IVA for binder samples after short-term and long-term aging. After RTFO and PAV aging, IVA remained the same for FSN-modified samples and control samples, which confirms that FSN have an advantageous effect on IVA. As can also be stated from Fig. 7c, increasing the aging time caused an increase in IVA. This effect can be explained by the change of chemical components and asphaltene content due to aging. An increase in viscosity is directly related to an increase of the asphaltene content^54^. Moreover, increasing the concentration of FSN in binder resulted in an increase in IVA, and in a reduction of aging resistance.

### Surface morphology

Figure 8a,b shows surface morphology of FSN in binder samples. Images of field emission scanning electron microscopy (FESEM) displayed that the FSN are well and uniformly dispersed (hexagonal structure shapes) in the binder matrix, and due to their large surface area the binder is stabilized.

Average particle sizes of FSN were calculated around 33 nm. The unique nanostructure shape of FSN in the binder matrix affects the aging process: FSN acts like a shield that prevents the upper structure from destruction, and traps volatile compounds, which decelerates their leaving process from binder^36^. Based on FESEM analysis, the silica nanoparticles in the binder surfaces have created uniform aggregates with average diameter of 100–300 nm.

FESEM images before and after aging are shown in Fig. 8c,d, where FSN appear in the form of bright dots in dark background (binder matrix). This Figure shows a comparison of the microstructure surface between the binder modified with FSN before and after aging. This change in topography of the binder surface may be related to the release and leave of some molecular groups from binder structure due to aging^55^.

### Microstructure observations

**Figure 9:**
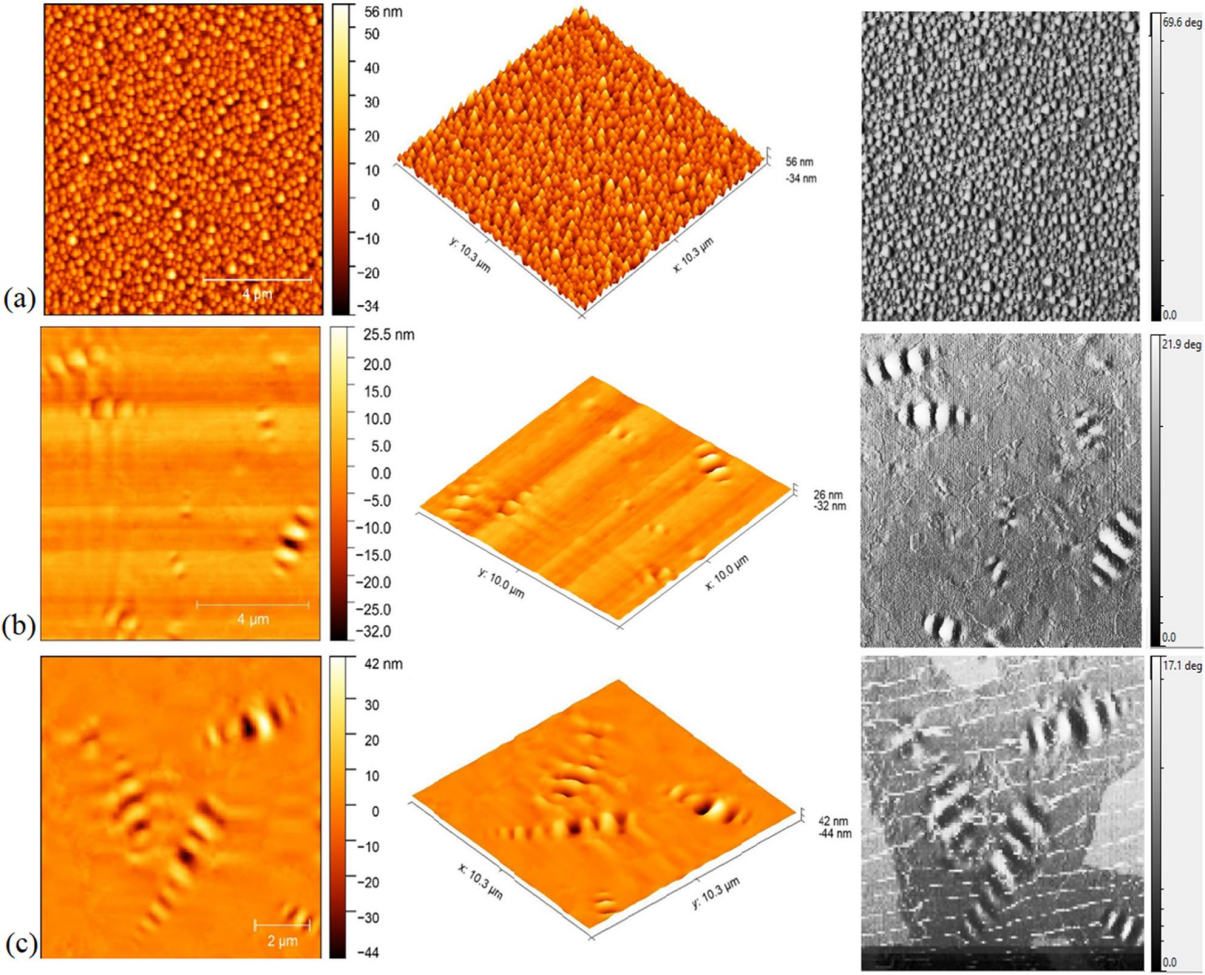
AFM morphology images of asphalt binder: (**a**) unaged, (**b**) short-term aged, and (**c**) long-term aged; at three different modes: right row) 2D topography, center row) 3D view of the surface, and left row) phase image.

In order to better understand the performance of FSN in un/aged binder, morphology and micro properties were investigated through AFM testing. The glass substrate was coated with binder sample via the method proposed by^56^. Identification of microstructures in binder is of interest because it illustrates any change of the chemical compounds and molecular interactions in the asphalt binder^57^. The microstructures of binder samples with FSN are shown in Fig. 9. In Fig. 9, typical catana phase (bee-like structures) are shown, surrounded by a peri phase (dispersed phase), and a para phase (smooth matrix phase). The matrix phase and the catana phase are usually considered as binder microstructure characteristic features^58^. According to previous studies “bee-like” structures could be the long alkyl chain asphaltenes and highly aromatics, and the microcrystalline waxes, which crystallize during cooling to the testing temperature^59^. Figure 9 present the Z-scale of all AFM microstructure images in the range of 60–90 nm. In the unaged asphalt binder (Fig. 9a), we found a network structure of spheres with no trace of bee structures. The main reasons that no bee structures are observed in the unaged asphalt binder relate to asphalt binder covering with a thin layer of FSN as well as no aging effect on asphalt binder. After short-term aging of asphalt binder (Fig. 9b), bee stripes (bee structures) appeared on the surface, associated with wax crystallization and long alkyl chain asphaltene^59^. In long-term aging (Fig. 9c), bee stripes become thinner on the surface which is due to the high polarity of asphalt binder molecules in comparison with short-term aging process. The comparison of AFM images of virgin and aged samples shows reduced heights and thicknesses of bee structures. These changes are due to the volatilization and polymerization of light components of binder during the aging process^60^. Also, the existence of light hydrocarbon molecules (> C10) within the long-term aging process can result in binder volatilization and the creation of a network of large molecules^60^. The addition of FSN to the binder provokes a change in the binder morphology and microstructure. The AFM images show that FSN causes create a support layer in nano scale in binder samples. These different observations with respect to studied conventional techniques for the binder microstructural properties as DSR, TGA, and contact angle provides AFM a valuable way to assess the topography of binder microstructures at the nano/microscopic level.

### Contact angle of asphalt binders

**Figure 10:**
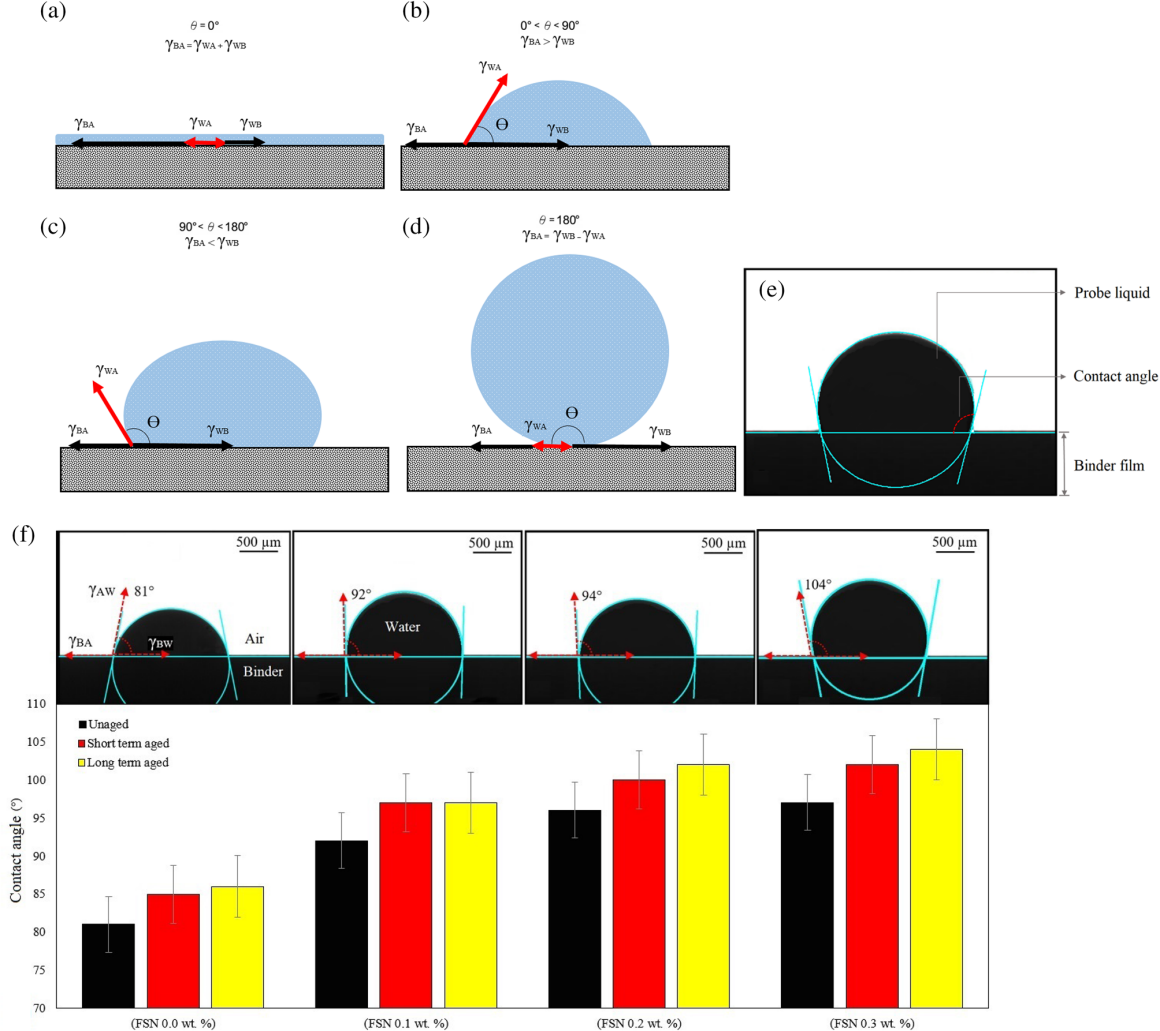
Schematic diagrams of different surface wettability situations: (**a**) absolute wetting (super hydrophilic), (**b**) high wetting, (**c**) low wetting, (**d**) absolute non-wetting (super hydrophobic), and (**e**) a liquid droplet on asphalt binder film. (**f**) Contact angles of modified binders in different concentrations of fumed silica nanoparticles. Interface energy of binder-water is denoted by γ_BW_, solid surface energy of binder-air by γ_BA_, liquid surface energy of air–water by γ_AW_.

The contact angle is the base of calculation of the surface free energy (SFE) which is the most important factor in the cohesive and adhesive of the asphalt mixture structure and is directly related to the moisture damage of asphalt pavement. The Contact Angle (CA) is a feature to quantify the wettability of solid surfaces when in contact with a droplet^61^. A CA value smaller or higher than 90° on a solid substrate shows a substrate is hydrophilic or hydrophobic, respectively. Wettability and liquid droplet on asphalt binder film with interphase energies is schematically shown in Fig. 10. With increasing CA value on the substrate, the water molecule that traps in the binder surface reduces significantly and the moisture susceptibility improves. By analyzing each CA image in detail (Fig. 10f), it can be seen that adding different concentrations of FSN in the asphalt binder leads to increased surface hydrophobicity of unaged, and short/long-term aged modify binders. This data, with FSN, suggests that the moisture susceptibility of asphalt mixture improved, even when adding a lower concentration of FSN (0.1 wt%) compared to the sample without FSN. The good moisture susceptibility of samples with FSN can be attributed to minimal surface adhesion energy of droplets on the solid substrate compared with the cohesive energy. AFM imaging also showed that an increased aging level could reduce the roughness of the binder surface and increase the contact angle.

## Mechanism

**Figure 11:**
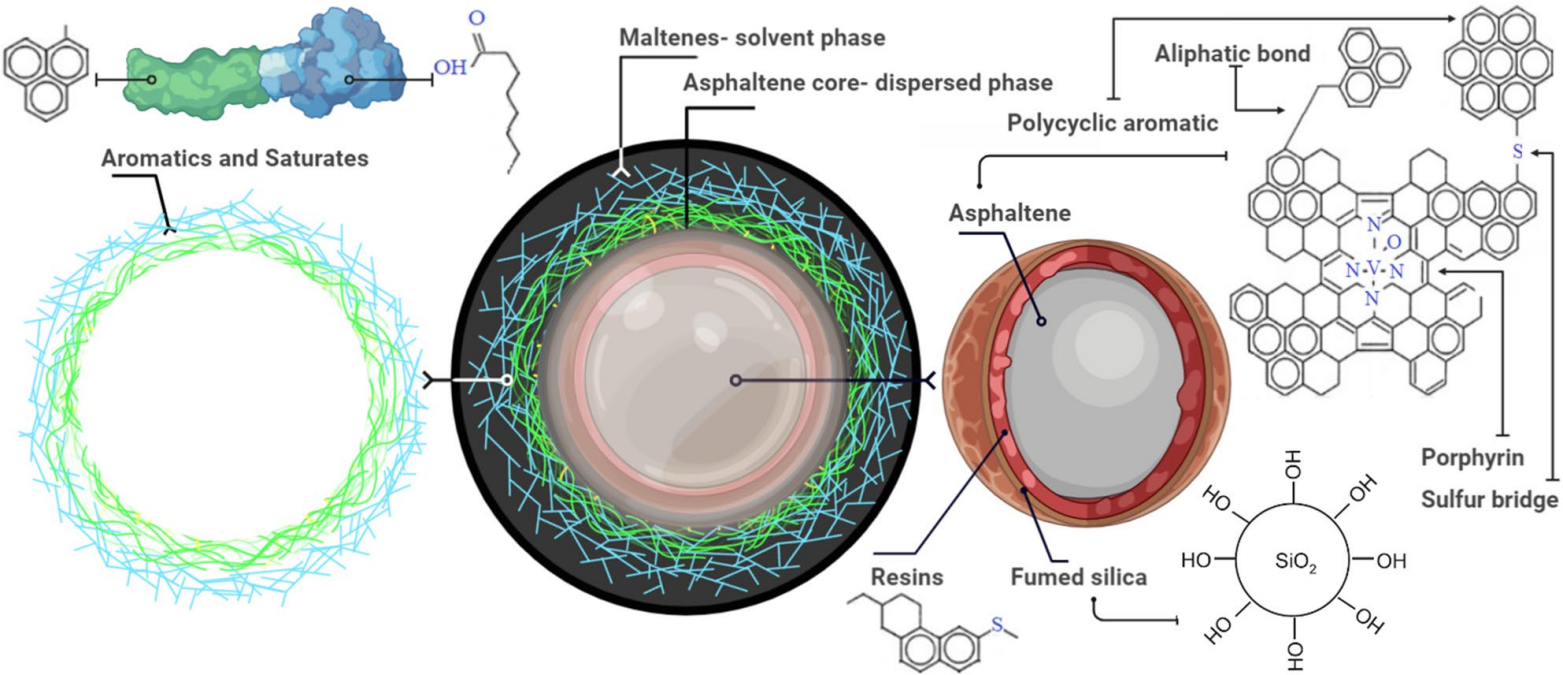
Schematic representation of the FSN action in the colloidal structure of bitumen.

There are several mechanisms that influence this phenomenon (improved aging asphalt binder by FSN). The initial subject is related to hydroxy groups bonding between binder and silica nanoparticles surface, which increase according to surface energy^62,63^. In addition, the high surface area to volume ratio (a unique feature) of FSN causes the combinability of binder molecules with fumed silica particles to increase^64^. Silica NPs could support binder molecules through chemical bonds and physical reaction (van der Waals force)^65^. Based on the colloidal structure of bitumen (Fig. 11), asphaltene and oil disperse during the solvent phase. Asphaltenes play a vital role in bitumen rheological properties. The average diameter of asphaltene particles is 0.5–40 nm^66,67^, and fumed silica nanoparticles (average particle size of silica particles is 33 nm) are suitable for chemical reaction and disperse between these colloidal dimensions. As shown in Fig. 11, the chemical characteristics of asphaltenes and aroma compounds can be supported by FSN, and therefore, mechanical and thermal properties are improved. Furthermore, nanoparticles of silica have superior abilities to change the surface properties of bitumen. They disperse in the colloidal structure of bitumen with nanoparticles at 1–100 nm scale, which hinders oxygen penetration to the bitumen matrix^68^. Some other chemical and physical properties of these nanoparticles such as ion exchange reactions and wettability alteration^69^ increase the stability of modified binder. However, excessive FSN may destroy the elastic properties of bitumen^70^. The aggregation of nanoparticles in binder matrix is related to Ostwald ripening and Van der Waals gravity^71^.

As for WMA technology, water molecules due to incomplete vaporization may become trapped in the asphalt mixture, eventually increasing moisture susceptibility, stripping, and premature breakdown of the asphalt pavement^5^. Based on the interfacial energy theory, molecules of water decrease the free surface energy of the asphalt aggregates and form a stable thermodynamically combination in a three-phase (water–binder–aggregate) system^72^. The silica NPs change the surface free energy and decrease the capillary inhibition between aggregate and water^73^. FSN leads to a distinct increase in contact angle, and hence, enhanced the hydrophobic property. In addition, according to chemical reaction theory, aggregate and binder create covalent weak bonds^74,75^. Molecules of water cause hydrolysis and the gradual decomposition of aggregate surfaces, and mechanism changes in pH-value of the molecules of water around the binder and aggregate^76^. This gradually alters the polar groups absorbed by the aggregates, and as a consequence, leads to separation of bitumen from the aggregate surface^75^. FSN, used in combination with the hydrophobic feature can reverse the condition.

## Discussion

In summary, a novel asphalt binder by fumed silica nanoparticles (FSN) has been developed which as an anti-aging binder in warm mix technology that can not only serve to reduce temperature but also overcome moisture susceptibility limitations. Morphological, chemical, and rheological properties of FSN-modified asphalt binder samples were screened, considering virgin and aged binder conditions, consistently compared with conventional non-modified asphalt binder samples. Based on the results, it is concluded that FSN may be successfully used as anti-aging agent in asphalt binders.

FSN modification improves the aging resistance of the asphalt binder, which was observed through various investigational techniques. A significant reduction in the index of viscosity aging was identified from rheological Dynamic Shear Rheometer (DSR) tests, which is calculated from the aged binder viscosity and the unaged binder viscosity. The three investigated concentrations of FSN in the asphalt binder displayed different effects on short-term and long-term aging resistance. Low concentrations of FSN reduced the stiffness level of the aged asphalt binder, improving performance. An optimum concentration of 0.1 wt.-% of FSN in the asphalt binder was identified.

DSR results indicated that FSN-modified asphalt binder is more resistant to rutting. Moreover, Bending Beam Rheometer results indicated that FSN-modified asphalt binder is more resistant to low-temperature cracking. The standard SHRP criterion to avoid low-temperature cracking was fulfilled when using 0.1 wt.-% of FSN in asphalt binder. FTIR (Fourier-Transform Infrared Spectroscopy) results also indicated that FSN acts as an anti-aging additive. After aging, the chemical aging index significantly decreased in FSN-modified asphalt binder samples. Investigations into binder surface morphology through AFM (Atomic Force Microscopy), showed distinct wrinkled bee structures of FSN-modified asphalt binder samples, which were even rougher after aging. It was concluded, that FSN-modification improves the usually observed negative influence of aging on asphalt micro-morphology and adhesion properties. From wettability analysis it was found that an increase in FSN content leads to a distinct increase in contact angle and reduction in trapping water molecules in the binder surface, and hence, to enhanced hydrophobic property, indicating that the moisture susceptibility of FSN-modified asphalt binders was improved.

Therefore, the presented research is interdisciplinary and covers existing gaps in asphalt technology. In addition, our findings on the concept of the molecular interaction between nanocomposite and asphalt binders can open new avenues for the application of nanotechnology in asphalt engineering.

## Methods

### Materials and methodology

In this study, FSN from Aerosil A200, Evonik, Germany, and bitumen 50/70 from Total, France were used. Fischer–Tropsch (FT) waxes (Sigma-Aldrich, Germany; Evonik, Germany; Sasol, South Africa) were used to modify the warm-mixture additive. Before use, FSN was dried in an oven at 110 °C for 3 h. In the first step, samples were prepared in accordance with prior works procedures^10,36^. Subsequently, FSN were added to bitumen in different values (0.1, 2 and 3 wt.-%). In this study bitumen modified by a 3% warm-mixture additive. This value was chosen based on the commonly used wax base content of WMA reported in previous researches^5,78^.

### Aging process

For RTFO test, the samples were kept at a temperature of 163 °C in the Rolling Thin-Film Oven (RTFO 8, model of ISL, France) according to ASTM D1754. Samples were long-term aged according to the PAV standard procedure (300 psi, 100 °C for 20 h). Samples were prepared in three conditions: sample unaged (S1-S4), samples aged in RTFO conditions (S5-S8), and samples aged in PAV conditions (S9-S12). The reference sample (S1, S5, S9) in this study was considered bitumen with WMA additive. An overview is presented in Table 2.

**Table 2:** Samples with different additives and aging processes.

Sample no.	Modification	Aging process	Sample no.	Modification	Aging process
S1	–	Unaged	**S7**	0.2 wt.-% FSN	RTFO
S2	0.1 wt.-% FSN	Unaged	**S8**	0.3 wt.-% FSN	RTFO
S3	0.2 wt.-% FSN	Unaged	**S9**	–	PAV
S4	0.3 wt.-% FSN	Unaged	**S10**	0.1 wt.-% FSN	PAV
S5	–	RTFO	**S11**	0.2 wt.-% FSN	PAV
S6	0.1 wt.-% FSN	RTFO	**S12**	0.3 wt.-% FSN	PAV

### Characterization methods

#### Rheological properties

A Dynamic Shear Rheometer (DSR, Kinexus DSR, UK) was used to evaluate binder linear viscoelastic properties under different boundary conditions (frequencies 1–2 Hz, and temperatures between 20 and 70 °C). The complex shear modulus (G*) and the phase angle of control binder and aged binder samples were measured in accordance with AASHTO T 315.

A thermoelectric Bending Beam Rheometer (TE-BBR, Cannon Ins., USA) was used to test low temperature flexural creep properties of the binder. The creep rate (m) and creep stiffness (S) of control samples and aged binder samples were measured based on AASHTO T 313.

#### Surface and thermal properties

Field Emission Scanning Electron Microscopy (FESEM) (TE-SCAN, MIRA III, Czech Republic) and Atomic Force Microscopy (AFM) (Nanowizard, JPK Ins., Germany) with cantilevers in tapping mode (RTESP, Bruker, USA) were used to investigate nano- and micro-structures of additives in binder samples. The substrate was coated with binder via the method proposed by Soenen et al^56^. Thickness and roughness map images at a set-point z and 1–2 frames s^−1^ were tested and then images (10 × 10 µm^2^) evaluated using the open-source software Gwyddion^77^_._ A thermal infrared camera (FLIR-T440, US) was recorded thermographic images binder samples over certain time periods.

#### Chemical and mechanical properties

Chemical properties were tested using Fourier Transform Infrared Spectroscopy (FTIR) (Thermoscientific Nicolet iS10, USA) and a Differential Scanning Calorimetry-Thermal Gravimetric Analysis (DSC–TGA) (SDT Q600, TA Ins., USA).

**Figure 12:**
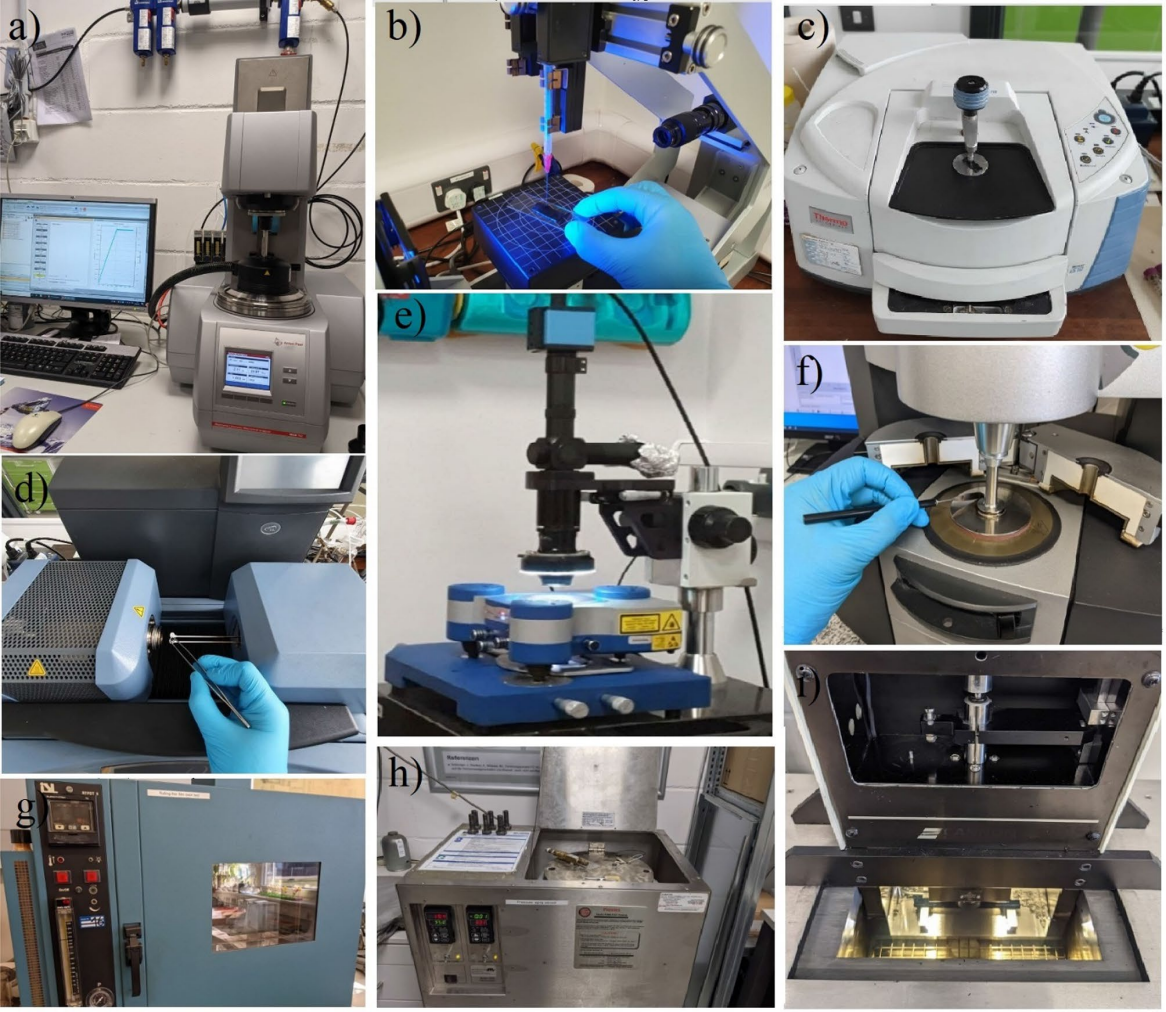
Devices used in the study: (**a**) rotational rheometer, (**b**) drop shape analyzer, (**c**) Fourier transform infrared spectroscopy, (**d**) thermal gravimetric analysis, (**e**) atomic force microscopy, (**f**) dynamic shear rheometer, (**g**) rolling thin-film oven, (**h**) pressure aging vessel, and (**i**) bending beam rheometer.

In addition, the mechanical properties of binder were measured by a Petrotest© machine (PK A5, Germany) for identifying softening point ring and ball according to ASTM D36 (2014), a digital ductilometer machine (Infratest, 20-2356, Germany) with a length of 1500 mm according to standard ASTM D113 (2007), and an Anton Paar automatic penetrometer (PNR 12, Germany) according to standard ASTM D5, (2013) to determine needle penetration. Table 3 summarizes physical properties of bitumen determined in this study. The contact angles (CA) were measured using a drop shape analyzer (DSA30, Krüss, Germany) by sessile drop method under room temperature. The morphologies were characterized by focusing an electron beam on surface of samples. Figure 12 shows the experimental devices used in the study.

**Table 3:** Physical properties of bitumen samples.

Physical properties	Ductility (@25 °C, cm)	Softening point (°C )	Penetration (@25 °C , 0.1 mm)	Density (kg/m^3^)
Value	100	48.6	63	1.03
Standard	ASTM D113	ASTM D36	ASTM D5	ASTM D70
